# Efficacy of platinum-based and non-platinum-based drugs on triple-negative breast cancer: meta-analysis

**DOI:** 10.1186/s40001-022-00839-0

**Published:** 2022-10-15

**Authors:** Canling Lin, Jiajun Cui, Zhen Peng, Kai Qian, Runwen Wu, Yimin Cheng, Weihua Yin

**Affiliations:** 1 College of Chemistry and Biological Engineering, Yichun University, Yichun, 33600 Jiangxi China; 2 Center for Translational Medicine, School of Medicine, Yichun University, Yichun, 33600 Jiangxi China; 3 Yichun People’s Hospital, Jiangxi Province, Yichun, 33600 Jiangxi China

**Keywords:** Carboplatin, Cisplatin, Triple-negative breast cancer, Neoadjuvant chemotherapy

## Abstract

**Background:**

Triple-negative breast cancer (TNBC), the subtype of breast cancer with the highest mortality rate, shows clinical characteristics of high heterogeneity, aggressiveness, easy recurrence, and poor prognosis, which is due to lack of expression of estrogen, progesterone receptor and human epidermal growth factor receptor 2. Currently, neoadjuvant chemotherapy (NAT) is still the major clinical treatment for triple-negative breast cancer. Chemotherapy drugs can be divided into platinum and non-platinum according to the presence of metal platinum ions in the structure. However, which kind is more suitable for treating TNBC remains to be determined.

**Methods:**

The relevant randomized clinical trials (RCTs) that explore the effectiveness of chemotherapy regimens containing platinum-based drugs (PB) or platinum-free drugs (PF) in treating TNBC patients were retrieved through PubMed, EMBASE, Cochrane Library, CNKI, and other literature platforms, above research findings, were included in the meta-analysis. The incidence of overall remission rate (ORR), pathological complete remission rate (pCR), overall survival (OS), disease-free survival (DFS), progression-free survival (PFS), and adverse events (AE) were compared between the two groups.

**Results:**

In this study, 12 clinical trials with a total of 4580 patients were included in the analysis. First, the ORR in 4 RCTs was, PB vs PF = 52% vs 48% (RR = 1.05, 95% CI: 0.91–1.21, *P* = 0.48); the pCR in 5 RCTs was, PB vs PF = 48% vs 41% (RR = 1.38, 95% CI: 0.88–2.16, *P* = 0.17). CI: 0.88–2.16, *P* = 0.17; the other 2 RCTs reported significantly higher DFS and OS rates in the PB group compared with the PF group, with the combined risk ratio for DFS in the PB group RR = 0.22 (95% CI:0.06–0.82, *P* = 0.015); the combined risk ratio for DFS in the PF group RR = 0.15 (95% CI. 0.04–0.61, *P* = 0.008); OS rate: PB vs PF = 0.046 vs 0.003; secondly, 2 RCTs showed that for patients with BRCA-mutated TNBC, the pCR rate in the PB and PF groups was 18% vs 26%, 95% CI: 2.4–4.2 vs 4.1–5.1; meanwhile, the median subject in the PB group The median PFS was 3.1 months (95% CI: 2.4–4.2) in the PB group and 4.4 months (95% CI: 4.1–5.1) in the PC group; finally, the results of the clinical adverse effects analysis showed that platinum-containing chemotherapy regimens significantly increased the incidence of adverse effects such as thrombocytopenia and diarrhea compared with non-platinum regimens, while the incidence of adverse effects such as vomiting, nausea, and neutropenia was reduced. The incidence of adverse reactions was reduced.

**Conclusion:**

Compared with non-platinum drugs, platinum drugs significantly improved clinical treatment effective indexes, such as PCR, ORR, PFS, DFS, and OS rate in the treatment of TNBC patients without BRCA mutant may cause more serious hematological adverse reactions. Accordingly, platinum-based chemotherapy should be provided for TNBC patients according to the patient's special details.

## Introduction

Triple-negative breast cancer (TNBC) is characterized by negative expression of estrogen (ER), progesterone receptor (PR), and human epidermal growth factor receptor 2 (HER2) [1]. Compared with other BCS, due to the lack of effective molecular targets for its clinical treatment, it is highly heterogeneous, aggressive, prone to recurrence, and has a poor prognosis. Based on the unsatisfactory effect of traditional hormone therapy on triple-negative breast cancer, the current standard treatment for TNBC patients includes neoadjuvant chemotherapy (NAT), surgery, and radiotherapy, of which NAT is the main treatment method.

For TNBC, the first-level NAT regimen is recommended in the Chinese breast cancer treatment guidelines, which include: (i) taxane, anthracycline, and cyclophosphamide; (ii) taxes anthracycline; (iii) taxane platinum. Among them, paclitaxel, as one of the commonly used drugs in the treatment of adenocarcinoma, can inhibit cell division by promoting tubulin polymerization and maintaining its stability, and finally induce cancer cell apoptosis [2]; anthracyclines are embedded in the form of reversibly binds to DNA double helix, affects DNA replication and unwinding, and interfering with the transcriptional process of tumor cells and preventing mRNA synthesis, thereby inhibiting the proliferation and spread of tumor cells [3]; cyclophosphamide belongs to nitrogen mustard drugs, which are metabolized by liver enzymes such as cytochrome P-450 to form active alkylating agents phosphor amide mustard gas and acrolein, phosphor amide metabolites in guanine N. The intra- and inter-strand adjacent DNA strands at the -7 position form cross-links and induce tumor cell apoptosis [4]; platinum drugs are commonly used in the treatment of ovarian cancer and breast cancer (especially BRCA1, BRCA2 gene-mutated triple-negative breast cancer), which affects DNA double-strand replication by forming intra-strand cross-links with tumor cell DNA and induces tumor cell apoptosis [5–7]. Existing studies have shown that platinum-based chemotherapy drugs combined with taxane-based chemotherapy drugs can synergistically inhibit the proliferation and spread of cancer cells, and at the same time can reduce the systemic toxicity caused by the drugs, cisplatin and carboplatin [8–10]. However, although platinum drugs have strong antitumor activity, their long-term overdose can induce a series of clinical adverse reactions, among which thrombocytopenia is the most common [11, 12], Adverse reactions such as myelosuppression are aggravated, so it should be carefully considered when long-term clinical use of this combination chemotherapy regimen is required. Finally, the evaluation indicators of the clinical treatment effect of breast cancer include survival rate, recurrence rate, remission rate, etc. Among them, the pathological complete remission rate PCR is an important indicator to measure the effect of NAT treatment. Few studies have found that the PCR of the PB regimen is higher than that of the PF. scheme [13–16]; on the other hand, the researchers found after a 56.2-month follow-up survey of patients: DFS of PB: PF was RR = 0.75 vs 0.62, *P* = 0.43; OS of PB: PF was RR = 0.90 vs 1.10, *P* = 0.58, the DFS of platinum-based therapy was significantly higher than that of platinum-free therapy, while the OS of patients treated with PB and PF was opposite [11, 17]. In conclusion, compared with non-platinum drugs, whether platinum-based drug-based therapy is more suitable for the treatment of TNBC patients needs to be further clarified.

In the clinical trials included in this meta-analysis, the platinum-based chemotherapy drugs were cisplatin and carboplatin, and the non-platinum-based chemotherapy drugs were anthracycline-containing, paclitaxel, and cyclophosphamide. Many studies were integrated, and rigorous randomized controlled trials were used to extract a certain number of samples for meta-analysis, to further demonstrate the efficacy of platinum drugs in the treatment of TNBC.

## Materials and methods

### Search strategy

First, the databases searched in this study include PubMed, EMBASE, Cochrane Library, CNKI, and the search terms are "triple-negative breast cancer", "neoadjuvant chemotherapy", "carboplatin", "cisplatin", "platinum"; secondly, the retrieval process is not limited by date and language, the retrieval date is as of May 25, 2022, and the retrieval content also includes conference papers related to breast cancer research, so as not to miss relevant research results that have not been published. The abstract and main content of the paper are analyzed and screened. The analysis of this study was done jointly by Lin, and Cheng, Qian, and the differences arising during the analysis were resolved through discussions with Cui, Yin. Analytical methods in this study were performed following PRISMA guidelines [18].

### Outcome indicators

Efficacy evaluation according to the new efficacy evaluation criteria for solid tumors: RECIST guidelines (version 1.1), is divided into complete remission (CR), partial remission (PR), stable disease (SD), and progressive disease (PD), with CR PR calculating the overall effective rate of ORR [19]. To clarify the difference in the efficacy of platinum-based and non-platinum-based treatment regimens for triple-negative breast cancer, this study used ORR and pathological complete response rate (PCR) as the main indicators, and overall survival rate (OS) and disease-free survival (DFS) as secondary indicators. In addition, this research further conducted a statistical analysis on the incidence of adverse reactions such as neutropenia, thrombocytopenia, nausea, vomiting, and diarrhea caused by the two treatment regimens.

### Data extraction

Extract the following data content from the research results included in the meta-analysis: author, country, publication time, the sample size of triple-negative breast cancer patients in the platinum-containing drug group and non-platinum drug group, patient age and characteristics, disease type, interventions (i.e., drugs, treatment regimens), and clinical outcomes, as shown in Table 1.

**Table 1:** Characteristics of the literatures included in the meta-analysis

Author	Year	Content	Country	Sample (PB/PF)	Age	Intervene
Pin Zhang [24]	2016	Full text	China	92 (48/44)	24–73	Cb + P
Maria Cristina Aguilar Martinez [14]	2015	Abstract	America	61 (30/31)	47	DDP + P + D
Andreas Schneeweiss [9]	2019	Full text	Germany	1906 (961/945)	48	P + M + Cb
Oleg Gluz [15]	2018	Full text	America	336 (182/154)	50	Cb + P
Nadine Tung [25]	2020	Full text	Israeli	117 (60/57)	24–73	DDP
Madoka Iwase [16]	2020	Full text	Japan	179 (88/91)	NR	Cb + P + CEF
Chen Yanyu [22]	2021	Full text	China	84 (42/42)	37–66/36–68	Cb + P
Ke Da Yu [8]	2022	Full text	China	647 (325/322)	51	Cb + P
Andrew Tutt [26]	2018	Full text	England	376 (188/188)	NR	Cb
Yang Pan [23]	2019	Full text	China	48 (24/24)	42.41 ± 9.20/43.05 ± 9.41	Cb + P
Ingrid A Mayer [10]	2021	Full text	America	308 (148/160)	27–72/26–76	Cb/DDP
Sibylle Loibl [13]	2019	Full text	America	634 (476/158)	≥ 18	Cb + P

NR, not reported; DDP, cis-platinum; D, doxorubicin; Cb, carboplatin; P, paclitaxel; M, myocet; CEF, cyclophosphamide + epirubicin + 5-fluorouracil

### Inclusion and exclusion criteria

Research reports must meet the following criteria to be included in the analysis: (i) randomized controlled trials; (ii) clinical study in triple-negative breast cancer patients receiving only neoadjuvant therapy; (iii) intervention measures: (1) platinum drug treatment group: platinum-based representative drugs carboplatin and cisplatin alone or in combination with other drugs for the treatment of TNBC patients; (2) non-platinum drug treatment group: TNBC patients were treated with platinum-free chemotherapy; (iv) clinical outcome indicators: PCR, DFS, OS, ORR, treatment effective rate (%) = partial remission rate, complete remission rate/total sample size. Exclusion criteria: (i) the research content partially or completely did not meet the inclusion criteria; (ii) there were duplicate works of literature, opinion papers, reviews, case reports, etc.; (iii) the clinical research subjects were patients with metastatic triple-negative breast cancer.

### Data analysis

A meta-analysis was performed using Review Manager (version 5.3.5; Cochrane, Oxford, UK) to calculate dichotomous variables by pooled odds ratios (RRs) with corresponding 95% confidence intervals (95% CI). The heterogeneity of the included studies was analyzed by the *X*^2^ test (the test level was *α* = 0.1) [20], and was quantified by *I*^2^: when *I*^2^ > 50%, *P* < 0.1, indicating that there is heterogeneity in the analysis results, otherwise, When *I*^2^ < 50%, *P* > 0.1, no heterogeneity. On the one hand, if the heterogeneity among the study results is not obvious, the fixed-effect model can be used for data analysis; on the other hand, if the heterogeneity among the studies is significant, three methods of subgroup analysis, and sensitivity analysis, and meta-regression can be used. To eliminate, if *I*^2^ > 50% after treatment, *P* < 0.1, indicating that the heterogeneity between the study results cannot be eliminated, a random effects model should be used [19]. The studies included in this analysis are all randomized controlled trials, so the combined risk ratio (RR) and the corresponding 95% confidence interval (CIs) can be selected as effect indicators to evaluate the overall efficacy of PB and PF treatment regimens [21]. The same method was used for the analysis of the incidence of adverse reactions in the PB and PF groups.

## Results

### The characteristics of the included studies

**Fig. 1:**
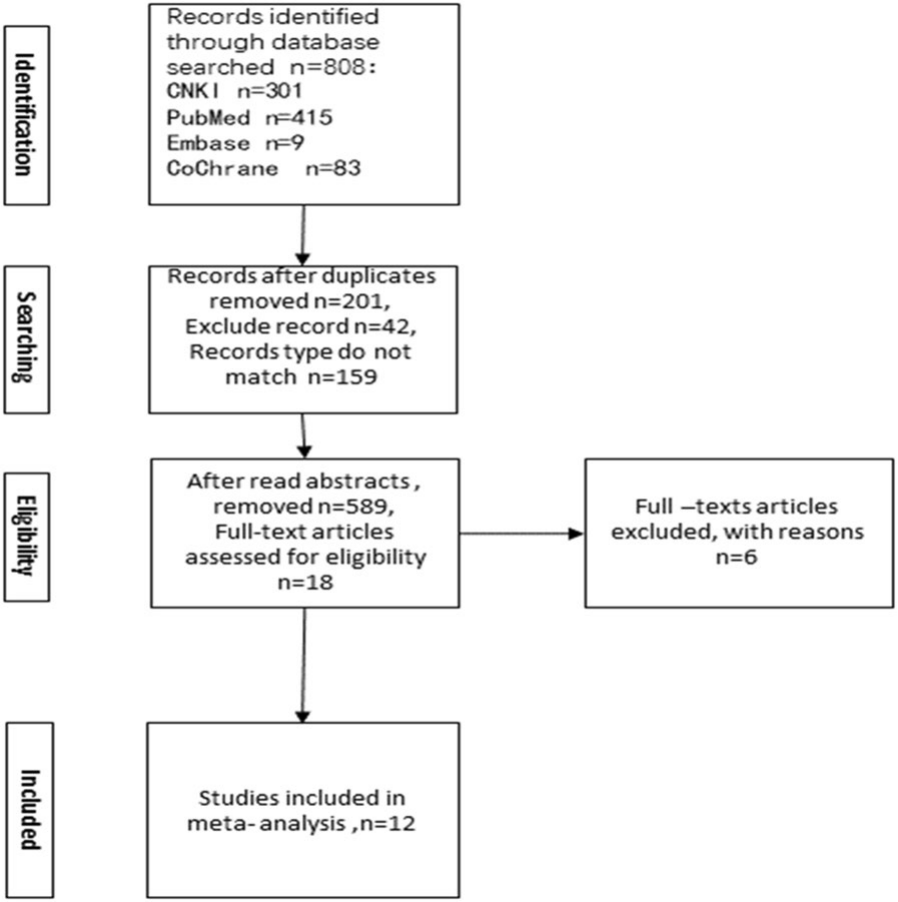
Flowchart of literature-related studies included in the meta-analysis

As shown in Fig. 1, 808 documents were retrieved through subject headings and keywords. The literature retrieve deadline was May 25, 2022. 42 duplicates were removed, 159 documents did not match the type of document, and 18 were left after full-text browsing, excluding the inconsistency of outcome indicators and control experiments, the full text was browsed again, and finally 12 literatures were included in the meta-analysis.

### Literature quality assessment

**Fig. 2:**
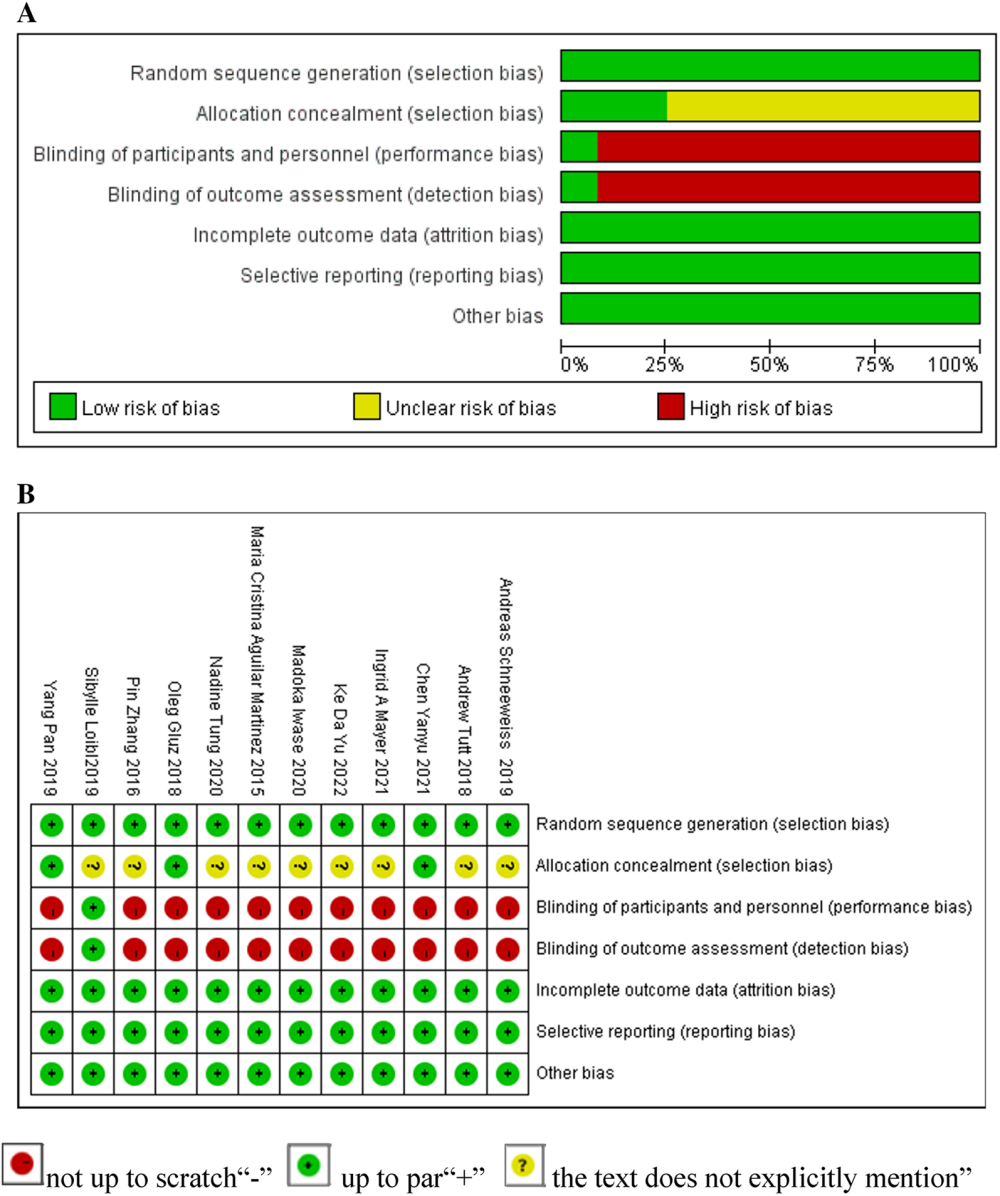
**A** Risk of bias graph: the authors' assessment of each item of risk of bias for the included literature as a percentage. **B** Overall risk of bias assessment: a review of the risk of bias item assessments included in the analysis

The overall and single literature quality of the included literature was rated by the Cochrane method of the British Center for Evidence-Based Medicine in 2001. It can be divided into three levels, with a total score of 6 points. The higher the score, the lower the risk of bias, and the higher the quality of the included literature [22]. As shown in Fig. 2A, the literatures included in the study can be divided into green (low risk), red (high risk), and yellow (unknown risk), and the quality evaluation criteria include the following seven items: random sequence generation (selection bias), allocation concealment (selection bias), participant blinding (performance bias), outcome assessment blinding (detection bias), incomplete outcome data (attrition bias), selective reporting (reporting bias), and other (bias due to vested financial interests and academic bias). Documents meeting only two levels were judged as low quality; documents meeting three levels or more were judged as high quality [22]. The literature is at low risk and has a suitable degree of matching. In addition, this study uses the same method to further analyze the risk of bias of the single literature included in the analysis, as shown in Fig. 2B, the standard is “ + ”, and the standard is not met “-”, and the text does not explicitly mention “?” [22]. Overall, the 12 included literatures have low risk of random sequence generation, incomplete result, data, selection bias, and other biases, and the total quality evaluation score is 6 points [23, 24], so the overall quality of the literature is high, which is at low risk.

### Contents of involved literature

The 8 clinical studies included in this meta-analysis involved > 100 subjects, and the other 4 subjects were < 100 subjects, with a total of 4580 patients, all aged ≥ 18 years, and platinum-based representative drugs were used. PB = 2452 subjects received neoadjuvant chemotherapy with cisplatin or carboplatin, and all of them received platinum-based NAT for the first time. Of the clinical studies included in this article, 6 used carboplatin combined with paclitaxel [8, 13, 15, 23–25], 3 used carboplatin or cisplatin alone [10, 26, 27], and the remaining 3 used NAT Carboplatin or cisplatin combined with other drugs; PF = 2128 subjects treated with non-platinum drugs, using a chemotherapy regimen of anthracycline, paclitaxel, fluorouracil, and cyclophosphamide.

#### The ORR rate

**Fig. 3:**
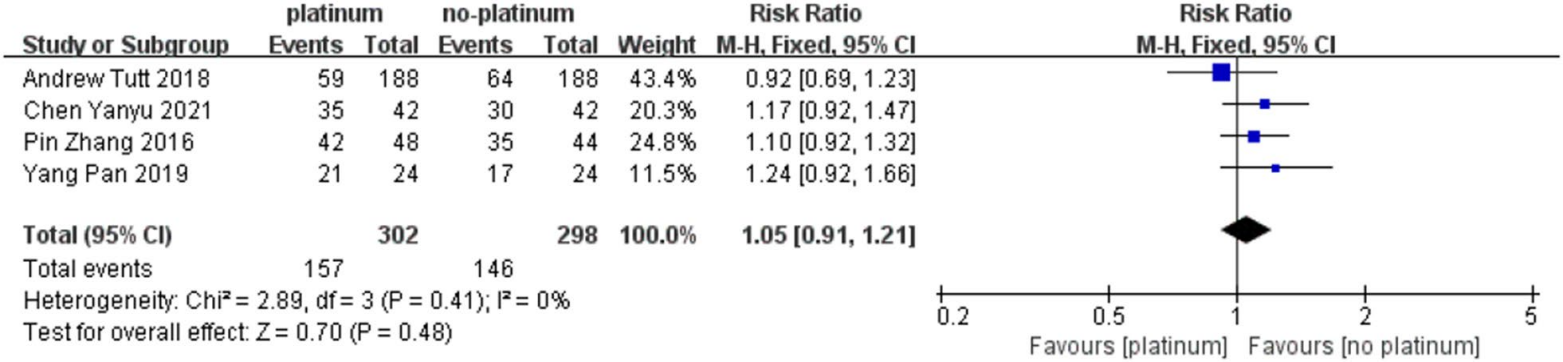
Forest plots representing pooled hazard ratios for ORR

Studies reported the ORR of 4 RCTs treated with platinum and non-platinum drugs for TNBC [23–25, 27], as shown in Fig. 3, after the heterogeneity test, the patients who received platinum drugs The overall response rate ORR of TNBC patients was significantly higher than that of non-platinum-treated patients, PB vs PF = 52% vs 48%, RR = 1.05, 95%CI: 0.91–1.21, I2 = 0%, *P* = 0.41, suggesting that There was no heterogeneity between the results of the different studies. To further ensure the accuracy and stability of the study, this study conducted a sensitivity analysis of the four included studies, and none of the studies caused great interference to this meta-analysis, so the fixed-effect model was further used to summarize the results of the above studies. Analysis showed RR = 1.05, 95%CI: 0.91–1.21, Z = 0.89, *P* = 0.48.

#### PCR rate

**Fig. 4:**
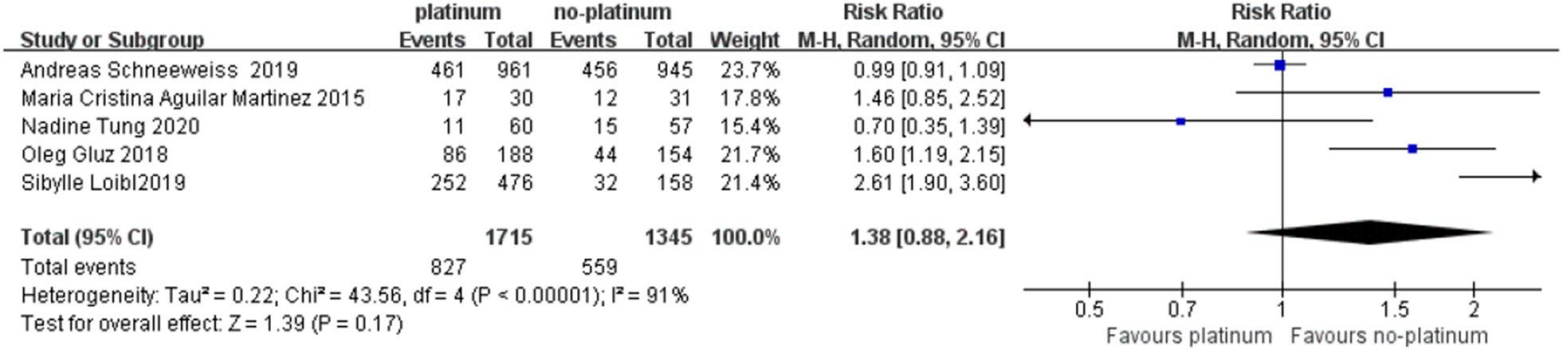
Forest plot for pooled hazard ratios for PCRs

Five studies have reported PCR rates for TNBC treated with platinum and non-platinum drugs [9, 13–15, 26], as shown in Fig. 4, there is high heterogeneity between the results of different study sexes, *I*^2^ = 91%, *P* < 0.00001. To clarify the source of heterogeneity, after sensitivity analysis and subgroup analysis of the 5 studies included in the analysis, no heterogeneity was found. Therefore, a random effects model was used to combine the effect size to conduct a pooled analysis of the PCR rates of the 5 studies: PB vs PF = 48% vs 41% (RR: 1.38, 95% CI: 0.88–2.16, *Z* = 1.39, *P* = 0.17), the PCR rate in the platinum-containing chemotherapy group was significantly higher than that in the non-platinum chemotherapy group. It is worth noting, however, that two RCTs reported that TNBC patients with BRCA1 and BRCA2 mutations were more efficacious than platinum-containing drugs in patients with BRCA1 and BRCA2, PCR rate: 26% vs 18% (95% CI: 4.1–5.1 vs 2.4–4.2) [26, 27].

#### DFS rate, OS rate, and PFS rate

In this study, the DFS rate, OS rate, and PFS rate of the included studies were further analyzed statistically to clarify the effect of platinum-based and non-platinum-based chemotherapy on the survival of TNBC patients. Difference in impact: first, 3 studies demonstrated significantly higher DFS in the PB group than in the PF group [8, 10, 16]. However, after (NAC), patients with triple-negative breast cancer (TNBC) had lower DFS than expected, PB vs PF DFS = 42% (95%CI: 30–53) vs 49% (95%CI: 39–59)) [10]; second, the study reported that adding platinum drugs to NCT could significantly improve OS, DFS, PB and PF in TNBC patients, respectively [DFS: RR = 0.22, 95%CI: 0.06–0.82, *P* = 0.015, OS: *P* = 0.046], and [DFS: RR = 0.15, 95% CI: 0.04–0.61, *P* = 0.008, OS: *P* = 0.003 [8, 16]. Finally, it is worth noting that for TNBC patients with BRCA gene mutations, the PFS rate in the platinum-based chemotherapy group was lower than that in the non-platinum group: the median PFS in the PB group was 3.1 months (95% CI: 2.4–4.2), and the median PFS in the PF group was 4.4 months (95% CI: 4.1–5.1) [26, 27].

#### Clinical adverse reactions and incidence rates

The common adverse reactions of patients in the platinum-based chemotherapy group and non-platinum-based chemotherapy group in the 12 clinical randomized controlled trials analyzed in this study include: neutropenia, nausea and vomiting, diarrhea, thrombocytopenia, and severe grade 3–4 adverse reactions. The incidence of each adverse reaction in different groups is classified and explained below.

##### Neutropenia

**Fig. 5:**
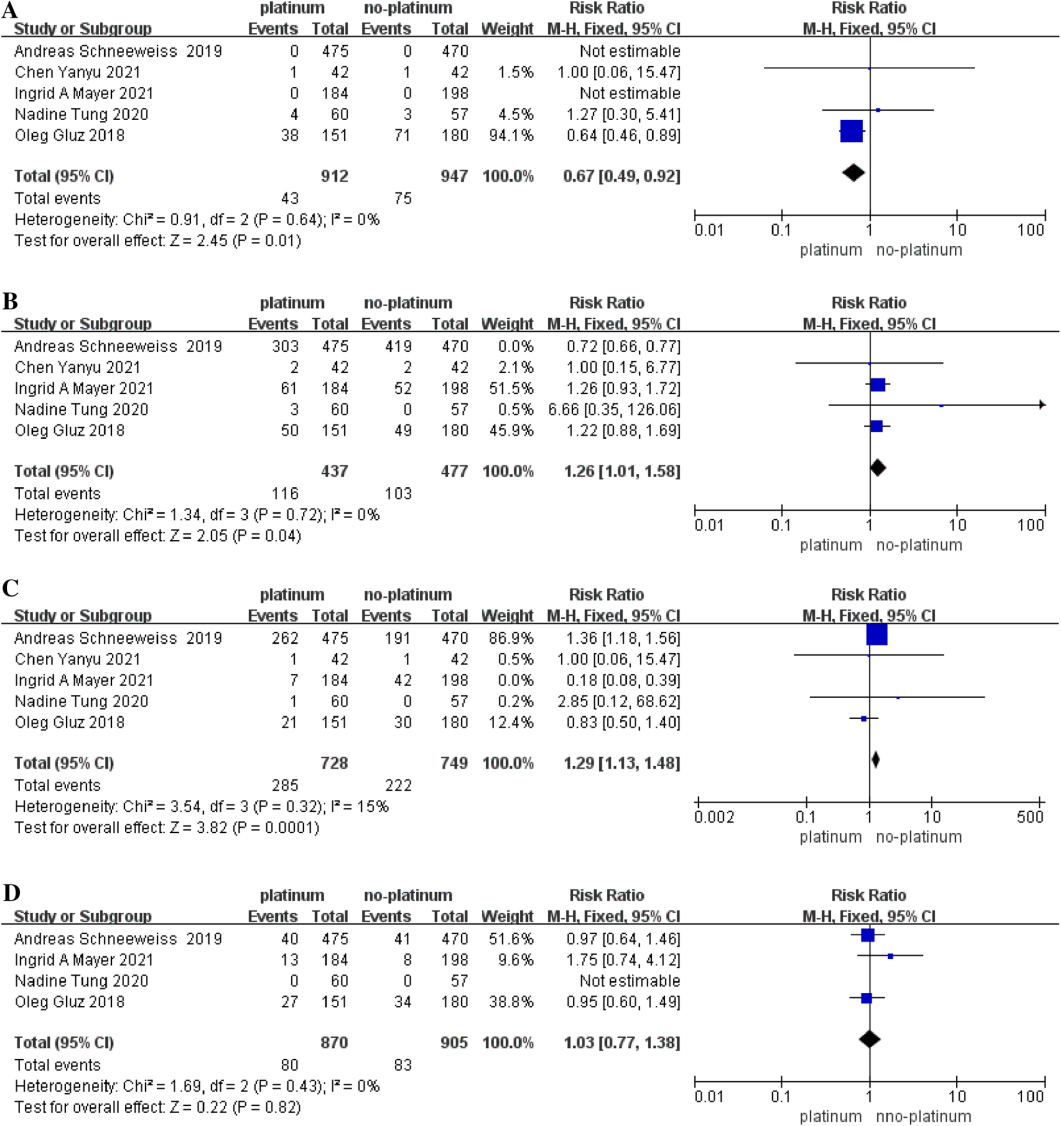
**A** Pooled hazard ratios for neutropenia. **B** Combined hazard ratio for nausea and vomiting. **C** The forest plot represents the pooled hazard ratio for developing diarrhea. **D** Forest plot of pooled hazard ratios for grade 3–4 adverse reactions

Five RCTs reported the incidence of neutropenia [9, 10, 15, 23, 26], of 1859 patients treated, 118 (6.3%) after neoadjuvant chemotherapy. Neutropenia (clinically manifested as the absolute value of neutrophils in peripheral blood less than 2.0 × 10^9^/L) occurred, of which 43 cases occurred in the platinum-containing chemotherapy group, and the other 75 cases occurred in the non-platinum drug chemotherapy group The incidence of neutropenia in the PB and PF groups was 4.7% and 7.9%, respectively (Fig. 5A).

##### Feel sick and vomit

Five RCTs have reported the incidence of nausea and vomiting [9, 10, 15, 23, 26]. Overall, 219 of 914 patients (24%) developed nausea and vomiting after treatment, including 116 of 437 patients in the PB group and 103 of 477 in the PF group. The probability of nausea and vomiting occurred. PB vs PF = 26.5% vs 21.6% (Fig. 5B).

##### Diarrhea

Diarrhea was reported in 5 randomized controlled trials [9, 10, 15, 23, 26], with 507 (34.3%) of 1477 patients developing diarrhea after neoadjuvant therapy, and 728 in the platinum-based chemotherapy group. There were 285 cases in the PB group and 222 out of 749 cases in the platinum-free chemotherapy group. The incidence of diarrhea was 39.1% in the PB group, which was significantly higher than that in the PF group (29.6%) (Fig. 5C).

##### Thrombocytopenia

Thrombocytopenia was reported in 1 RCT [15], 112 of 313 patients in this study (35.8%) had thrombocytopenia after NAT, and 100 of 151 subjects in the platinum-based chemotherapy group. For example, 12 adverse reactions occurred in the non-platinum chemotherapy group, and for this adverse reaction, PB vs PF = 66.2% vs 6.7%.

##### Grade 3–4 serious adverse reactions

Four RCTs reported the incidence of grade 3–4 adverse reactions [9, 10, 15, 26], and overall, 163 of 1775 patients (9.2%) experienced grades 3 and 4 adverse reactions, 80 out of 870 cases in the platinum chemotherapy group and 83 out of 905 cases in the non-platinum chemotherapy group, so there was no difference in the incidence of grade 3–4 adverse reactions in the PB and PC groups, both being 9.2% (Fig. 5D).

In conclusion, compared with non-platinum-based chemotherapy regimens, the incidence of adverse reactions such as thrombocytopenia and diarrhea caused by platinum-based chemotherapy significantly increased, while the incidence of adverse reactions such as vomiting, nausea, and neutropenia decreased.

#### Publication bias

**Fig. 6:**
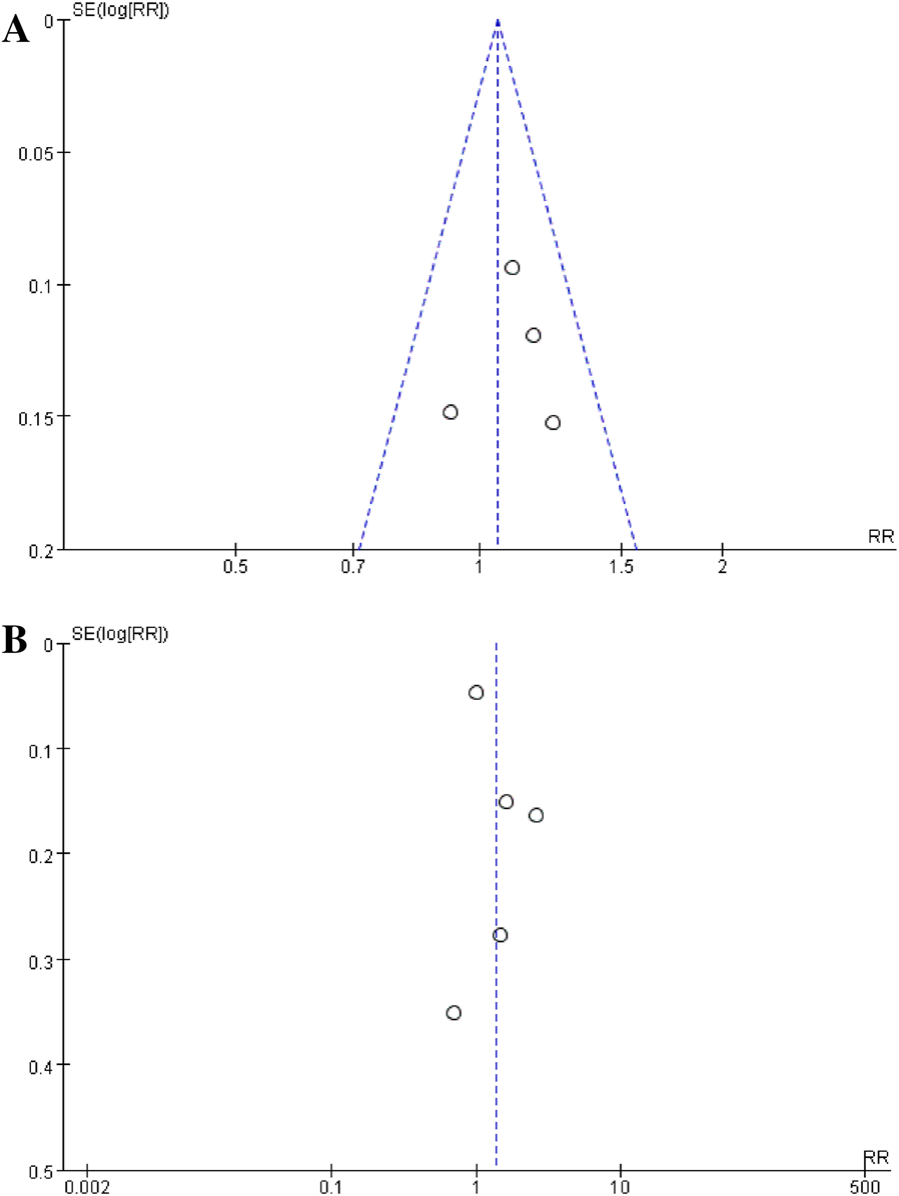
**A** Funnel plot of ORR rate. **B** Funnel plot of PCR rates

As shown in Fig. 6A, the funnel plot is asymmetric, and there is obvious publication bias; Fig. 6B. After sensitivity analysis and subgroup analysis, no source of heterogeneity was found, and there was publication bias.

## Discussion

In general, TNBC, a kind of breast cancer that the absence of HR and HER2 receptor expression renders endocrine or anti-HER2 targeted therapy ineffective, can be divided into multiple subtypes by profiling gene expression [28]. Similar to other tumors, classification of molecular subtypes of TNBC paves the way for molecular-targeted drug development, which would provide more options for clinical treatment [28, 29]. As a vital target for immunotherapy of various tumors, programmed cell death protein 1 (PD-1) and its ligand PD-L1 has a significant role in TNBC progression as well [30, 31]. In previous study [30], patient with high PD-L1 expression had been treated with pembrolizumab, a selective inhibitor of PD-L1. In contrast, if PD-L1 could not be detected, then chemotherapy is essentially the sole therapeutic option for TNBC. Neoadjuvant chemotherapy regimens for TNBC have been employed in various clinical studies in recent years, all of which have had some degree of success [32]. The above-mentioned research has demonstrated that, while neoadjuvant chemotherapy regimens may have many negative consequences, platinum combined with taxes can aid to exhibit their synergistic benefits in the treatment of breast cancer. On the one hand: compared with patients of no platinum scheme, which can upgrade ORR, PCR, DFS, and OS rates prominently. The study demonstrated that TNBC was closely related to *BRCA1* mutations, compared to patients with *BRCA2* mutations carriers or non-carriers, breast cancer patients with *BRCA1* mutation are more likely to have TNBC [33]. Compounds containing platinum display good antineoplastic activity to patients with TNBC, those with *BRCA* mutations, and the toxicity of agents is weak, patients can endure well, moreover, under prognostic exist circumstance, who had higher DFS, OS rates compared to patients who were without platinum [26, 27]. Besides, it was worth noting that as to TNBC patient carried *BRCA* mutation, poly-adenosine diphosphate ribose (ADP) polymerase inhibitors (PARPi) were preferred and then sacituzumab govitecan or other novel antibody drug conjugates (ADCs) or checkpoint inhibitors could be taken into account [34, 35]; On the other hand, according to various metabolic ways, that combination therapy, as above, can decrease bodily drug toxicity, consequently, platinum combined with paclitaxel had an opportunity to be the ideal scheme for curing TNBC [26, 27, 36].

Nevertheless, noticeably, the effect of adding platinum agents to different types of patients is still in dispute. At first, after conducting NAT, some people who didn’t reach the PCR rate, and were diagnosed with TNBC and RD, which were treated with platinum-based, whose survival situation was worse than patients with non-platinum. After conducting follow for around 3 years, found that a DFS rate of 42% (95% CI, 30–53) was achieved in patients who received platinum-based treatment, however capecitabine was 49% (95% CI, 39–59), which was lower than expected effect [10]; Secondly, the addition of platinum agents to NAT for TNBC patients with early-stage did not exhibit a large survival rate. Thirdly, it’s strange that, for BRCA mutation carriers in the I–III stage, who coexist with TNBC and ER^+^/HER2^−^ disease and advanced TNBC patients, the PCR and ORR rate of a group of PB did not higher than the group of PF [26, 27]. It is hereby that the people above inadvisable to choose platinum-based treatment [8, 11]. Lastly, the first report concerning real-world data on the Chilean population covered that females of TNBC in the phase of I–III received NAT of contained platinum. The result showed that the PCR rate was associated with extended overall survival, invasiveness, and disease-free survival, however whether the Cb exists or not was irrelevant to variability in survival indicators [37]. Similarly, other 2 studies suggest that whether patients of TNBC use platinum agents or not was related to PCR rate, which can induce the incidence of adverse reactions in the blood system increased markedly, such as febrile neutropenia [38, 39].

Based on the above controversy, this paper inclusively searched Chinese and English databases, the efficacy of PB compared with that of PB in the treatment of TNBC. This study included 12 trials of the group of based platinum (PB) and the group of non-platinum (PF), and select some indicators analysis, such as objective response rate (ORR), pathological complete response (PCR), overall survival (OS), disease-free survival (DFS), progression-free survival **(**PFS), adverse event (AE). Then, draw some conclusions: (i) among 4 RCTs, the ORR of the PB group (52%) was significantly higher than that of the PF group; (ii) in the 5 RCTs, the PCR of the two groups revealed significantly difference, as well, PB vs PF = 48% vs 41%, the PCR rate of PB group is significant higher than PF group. However, another 2 RCTs of TNBC patients with *BRCA* mutations, found that the PCR rate of the PB group and the PF group was 18%, and 26%, respectively, and the median PFS of the PB group was 3.1 months, while the median PFS of the PF group was 4.4 months. The above-mentioned results suggest that mutations of the *BRCA* gene in TNBC patients may affect the treatment effect of different therapy methods. Reckoning that, at present, only a few clinical studies have reported the efficacy of different therapies in patients with *BRCA* mutation TNBC, in the future, the interrelation and specific molecular mechanisms between the efficacy of *BRCA* and NAT need further exploration; (iii) besides PCR, the DFS and OS of the PB group and the PF group also exist significant difference: the combined hazard ratio of DFS in the PB group was significantly higher than that in the RR group by RR = 0.22; similarly, the OS rate (0.046) in the PB group was significantly greater than that in the PF group (0.003); (iv) the results of the clinical adverse reaction analysis revealed that: compared to non-platinum, chemotherapy regimens containing platinum compounds can significantly increase the incidence of adverse reactions such as thrombocytopenia and diarrhea, adversely, the incidence of adverse reactions such as vomiting, nausea, and neutropenia is reduced. In a word, in a clinic, we should consider all kinds of situations in patients synthetically, then, weigh the pros and cons of adding platinum agents into NAT, and during the treatment, follow-up regularly. The above-mentioned measures were vital to reducing patients’ clinical adverse reactions.

Notwithstanding, we need to clarify that there are several limitations in this meta-analysis: (i) due to the included samples being different-sized, may be existing publish bias; (ii) this study has quality problems, on account a majority of incorporated papers were not adopt blind; (iii) the mass of the studies involve in the present analyses lack follow-up data, such as disease-free survival (DFS), life quality. Nonetheless, these limitations haven’t affected the conclusion. Results from this study will provide some meaningful information for a controversial problem, which is about adding cisplatin into NAT, in TNBC patients, and assisting clinicians and patients in making optimal remedy decisions.

Overall, outcomes of 12 clinical trials were included in this review, according to a meta-analysis of it, concluding that platinum-based chemotherapy can significantly improve the PCR rate and prognosis in TNBC patients. We recommend that can be used as an alternative therapy by NAT. Although this study has certain limitations, such as without use of blind, different-sized samples, included studies were RCTs. Thus, the outcome had much more credibility, and further demonstrated that the efficiency of the addition of platins in the TNBC was higher than in non-platinum methods. Compared to most chemotherapy drugs that possess side effects, platinum-based treatment was lower. In future, more relevant clinical studies are required to verify the therapeutic effect of drugs acting on novel molecular biomarkers that target TNBC; meanwhile, more attention should be given to adverse reactions of clinical medication.

## Data Availability

Not applicable.

## References

[1] Liedtke C, Mazouni C, Hess KR (2008). Response to neoadjuvant therapy and long-term survival in patients with triple-negative breast cancer. J Clin Oncol.

[2] Kampan NC, Madondo MT, McNally OM (2015). Paclitaxel and its evolving role in the management of ovarian cancer. Biomed Res Int.

[3] 王西礼. 表柔比星联合新辅助化疗治疗三阴性乳腺癌的最佳剂量讨论. 中国合理用药探索, 2019, 16(06): 146–9.

[4] Ogino MH, Tadi P. Cyclophosphamide [M]. StatPearls. Treasure Island (FL); StatPearls Publishing LLC. 2022.

[5] Bhagat A, Kleinerman ES (2020). Anthracycline-induced cardiotoxicity: causes, mechanisms, and prevention. Adv Exp Med Biol.

[6] Wolff AC, Blackford AL, Visvanathan K (2015). Risk of marrow neoplasms after adjuvant breast cancer therapy: the national comprehensive cancer network experience. J Clin Oncol.

[7] 胡利敏, 陈述政, 周毅, et al. 密集AC序贯密集紫杉醇新辅助化疗对三阴性乳腺癌的近期疗效. 肿瘤学杂志, 2018, 24(03): 199–202.

[8] Yu KD, Ye FG, He M (2020). Effect of adjuvant paclitaxel and carboplatin on survival in women with triple-negative breast cancer: a phase 3 randomized clinical trial. JAMA Oncol.

[9] Schneeweiss A, Möbus V, Tesch H (2019). Intense dose-dense epirubicin, paclitaxel, cyclophosphamide versus weekly paclitaxel, liposomal doxorubicin (plus carboplatin in triple-negative breast cancer) for neoadjuvant treatment of high-risk early breast cancer (GeparOcto-GBG 84): a randomised phase III trial. Eur J Cancer.

[10] Mayer IA, Zhao F, Arteaga CL (2021). Randomized Phase III postoperative trial of platinum-based chemotherapy versus capecitabine in patients with residual triple-negative breast cancer following neoadjuvant chemotherapy: ECOG-ACRIN EA1131. J Clin Oncol.

[11] Saleh RR, Nadler MB, Desnoyers A (2021). Platinum-based chemotherapy in early-stage triple negative breast cancer: A meta-analysis. Cancer Treat Rev.

[12] Pathak N, Sharma A, Elavarasi A (2022). Moment of truth-adding carboplatin to neoadjuvant/adjuvant chemotherapy in triple negative breast cancer improves overall survival: An individual participant data and trial-level Meta-analysis. Breast.

[13] Loibl S, O'Shaughnessy J, Untch M (2019). Addition of the PARP inhibitor veliparib plus carboplatin or carboplatin alone to standard neoadjuvant chemotherapy in triple-negative breast cancer (BrighTNess): a randomised, phase 3 trial. Lancet Oncol.

[14] Martinez MCA, Arce-Salinas C, Alvarado-Miranda A (2015). Randomized phase II trial to evaluate the safety and efficacy of neoadjuvant cisplatin in combination with taxanes-anthracyclines vs taxanes-anthracyclines alone in locally advanced triple negative breast cancer. J Clin Oncol.

[15] Gluz O, Nitz U, Liedtke C (2018). Comparison of Neoadjuvant Nab-Paclitaxel+Carboplatin vs Nab-Paclitaxel+Gemcitabine in triple-negative breast cancer: randomized WSG-ADAPT-TN Trial Results. J Natl Cancer Inst.

[16] Iwase M, Ando M, Aogi K (2020). Long-term survival analysis of addition of carboplatin to neoadjuvant chemotherapy in HER2-negative breast cancer. Breast Cancer Res Treat.

[17] Sharma P, López-Tarruella S, García-Saenz JA (2018). Pathological response and survival in triple-negative breast cancer following neoadjuvant carboplatin plus docetaxel. Clin Cancer Res.

[18] Moher D, Liberati A, Tetzlaff J (2009). Preferred reporting items for systematic reviews and meta-analyses: the PRISMA statement. BMJ.

[19] Eisenhauer EA, Therasse P, Bogaerts J (2009). New response evaluation criteria in solid tumours: revised RECIST guideline (version 1.1). Eur J Cancer.

[20] 李文臣, 高歌, 伦志军, et al. 医学Meta分析中异质性的识别及处理方法, 斯里兰卡科伦坡, F, 2018 [C].

[21] Sargeant JM, O'Connor AM, Gardner IA (2010). The REFLECT statement: reporting guidelines for randomized controlled trials in livestock and food safety: explanation and elaboration. J Food Prot.

[22] Cumpston M, Li T, Page M J, et al. Updated guidance for trusted systematic reviews: a new edition of the Cochrane Handbook for Systematic Reviews of Interventions. Cochrane Database System Rev, 2019;10: 000142. 10.1002/14651858.ED000142 PMC10284251 31643080

[23] 陈艳宇, 傅思莹, 赵文珍, et al. 紫杉醇联合卡铂方案在三阴性乳腺癌新辅助化疗中的治疗效果及对患者病理变化的影响. 中国妇幼保健, 2021, 36(07): 1477–80.

[24] 杨盼. 紫杉醇联合卡铂辅助化疗治疗三阴性乳腺癌患者临床研究. 现代诊断与治疗, 2019, 30(08): 1227–8.

[25] Zhang P, Yin Y, Mo H (2016). Better pathologic complete response and relapse-free survival after carboplatin plus paclitaxel compared with epirubicin plus paclitaxel as neoadjuvant chemotherapy for locally advanced triple-negative breast cancer: a randomized phase 2 trial. Oncotarget.

[26] Tung N, Arun B, Hacker MR (2020). TBCRC 031: Randomized Phase II Study of neoadjuvant cisplatin versus doxorubicin-cyclophosphamide in germline BRCA Carriers With HER2-Negative Breast Cancer (the INFORM trial). J Clin Oncol.

[27] Tutt A, Tovey H, Cheang MCU (2018). Carboplatin in BRCA1/2-mutated and triple-negative breast cancer BRCAness subgroups: the TNT Trial. Nat Med.

[28] Lehmann BD, Bauer JA, Chen X, Sanders ME, Chakravarthy AB, Shyr Y, Pietenpol JA (2011). Identification of human triple-negative breast cancer subtypes and preclinical models for selection of targeted therapies. J Clin Investig.

[29] Iacovino ML, Miceli CC, De Felice M, Barone B, Pompella L, Chiancone F, Di Zazzo E, Tirino G, Della Corte CM, Imbimbo C, De Vita F, Crocetto F (2022). Novel therapeutic opportunities in neoadjuvant setting in urothelial cancers: a new horizon opened by molecular classification and immune checkpoint inhibitors. Int J Mol Sci.

[30] Heimes AS, Schmidt M (2019). Atezolizumab for the treatment of triple-negative breast cancer. Expert Opin Investig Drugs.

[31] Miles D, Gligorov J, André F, Cameron D, Schneeweiss A, Barrios C, Xu B, Wardley A, Kaen D, Andrade L (2021). Primary results from IMpassion131, a double-blind, placebo-controlled, randomised phase III trial of first-line paclitaxel with or without atezolizumab for unresectable locally advanced/metastatic triple-negative breast cancer. Ann Oncol.

[32] Luo S P, Wu Q S, Chen H, et al. Validation of the Prognostic Significance of the Prognostic Stage Group According to the Eighth Edition of American Cancer Joint Committee on Cancer Staging System in Triple-Negative Breast Cancer: An Analysis From Surveillance, Epidemiology, and End Results 18 Database. J Surg Res. 2020; 247: 211–9. 10.1016/j.jss.2019.09.072 31706539

[33] Chen H, Wu J, Zhang Z (2018). Association Between BRCA status and triple-negative breast cancer: a meta-analysis. Front Pharmacol.

[34] Robson M, Im SA, Senkus E, Xu B, Domchek SM, Masuda N, Delaloge S, Li W, Tung N, Armstrong A (2017). Olaparib for metastatic breast cancer in patients with a germline BRCA Mutation. N Engl J Med.

[35] Ledermann JA, Harter P, Gourley C, Friedlander M, Vergote I, Rustin G, Scott C, Meier W, Shapira-Frommer R, Safra T (2016). Overall survival in patients with platinum-sensitive recurrent serous ovarian cancer receiving olaparib maintenance monotherapy: An updated analysis from a randomised, placebo-controlled, double-blind, phase 2 trial. Lancet Oncol.

[36] Tian H, Ma D, Tan X (2021). Platinum and taxane based adjuvant and neoadjuvant chemotherapy in early triple-negative breast cancer: a narrative review. Front Pharmacol.

[37] Walbaum B, Acevedo F, Medina L (2021). Pathological complete response to neoadjuvant chemotherapy, but not the addition of carboplatin, is associated with improved survival in Chilean triple negative breast cancer patients: a report of real world data. Ecancermedicalscience.

[38] Sella T, Galyam EN, Levanon K (2018). Evaluation of tolerability and efficacy of incorporating carboplatin in neoadjuvant anthracycline and taxane based therapy in a BRCA1 enriched triple-negative breast cancer cohort. Breast.

[39] Fontaine C, Renard V, van den Bulk H (2019). Weekly carboplatin plus neoadjuvant anthracycline-taxane-based regimen in early triple-negative breast cancer: a prospective phase II trial by the Breast Cancer Task Force of the Belgian Society of Medical Oncology (BSMO). Breast Cancer Res Treat.

